# ESMO Congress 2021: highlights from the EORTC gastrointestinal tract cancer group’s perspective

**DOI:** 10.1016/j.esmoop.2022.100392

**Published:** 2022-02-16

**Authors:** T. Koessler, M. Alsina, D. Arnold, I. Ben-Aharon, M. Collienne, M.P. Lutz, C. Neuzillet, R. Obermannova, M. Peeters, F. Sclafani, E. Smyth, J.W. Valle, A.D. Wagner, L. Wyrwicz, E. Fontana, M. Moehler

**Affiliations:** 1 Department of Oncology, Geneva University Hospital, Geneva, Switzerland; 2 Swiss Cancer Center Leman (SCCL), University of Geneva, Lausanne, Switzerland; 3 Hospital Universitario de Navarra (HUN), Medical Oncology Department, Pamplona, Spain; 4 Vall d’Hebron Institute of Oncology (VHIO), Barcelona, Spain; 5 Department of Oncology, Haematology and Palliative Care, Asklepios Klinik Altona, Asklepios Tumorzentrum Hamburg, Hamburg, Germany; 6 Division of Oncology, Rambam Health Care Campus, Rappaport Faculty of Medicine, Technion, Haifa, Israel; 7 European Organisation for Research and Treatment of Cancer, Brussels, Belgium; 8 Caritasklinikum, Saarbrucken, Germany; 9 GI Oncology, Medical Oncology Department, Institut Curie Saint-Cloud, Versailles Saint Quentin University, Saint-Cloud, France; 10 Department of Comprehensive Cancer Care, Masaryk Memorial Cancer Institute and Faculty of Medicine, Masaryk University, Brno; 11 Department of Pharmacology, Faculty of Medicine, Masaryk University, Brno, Czech Republic; 12 Department of Oncology, Universitair Ziekenhuis Antwerpen, Antwerp, Belgium; 13 Department of Medical Oncology, Institut Jules Bordet-Université Libre de Bruxelles (ULB), Brussels, Belgium; 14 Cambridge University Hospitals NHS Foundation Trust, Addenbrooke’s Hospital, Cambridge, UK; 15 Division of Cancer Sciences, University of Manchester, Manchester, UK; 16 Department of Medical Oncology, The Christie NHS Foundation Trust, Manchester, UK; 17 Department of Oncology, Division of Medical Oncology, Lausanne University Hospital (CHUV) and University of Lausanne (UNIL), Lausanne, Switzerland; 18 Maria Sklodowska-Curie Memorial Cancer Center and Institute of Oncology, Warsaw, Poland; 19 Sarah Cannon Research Institute, London, UK; 20 Department of Internal Medicine, Johannes-Gutenberg University, Mainz, Germany

**Keywords:** oesophagus cancer, stomach cancer, pancreatic cancer, neuroendocrine tumours, colorectal cancer

## Abstract

There has been no major change of practice in gastrointestinal oncology at the European Society for Medical Oncology (ESMO) symposium 2021, but confirmation that immunotherapy in combination with chemotherapy has become standard of care in several indications. The European Organisation for Research and Treatment of Cancer (EORTC) Gastrointestinal Track Cancer Group has selected important phase II and III trials presented during the symposium across all gastrointestinal cancers as well as early reports on new drugs or new combinations that may change practice in the future.

## Highlights

• CheckMate 649 shows OS benefit from adding nivolumab to chemotherapy in advanced esophagogastric adenocarcinoma.
• Update from PRODIGE 24 study confirm OS superiority of modified FOLFIRINOX over gemcitabine in resected pancreatic cancer.
• Sunitinib improves PFS in progressive phaeochromocytoma and paragangliomas in the FIRSTMAPP study compared with placebo.

## Upper gastrointestinal tract

### EORTC Task force Oesophagus and Stomach, chairs: Elizabeth Smyth, Anna Dorothea Wagner

At this years’ European Society for Medical Oncology (ESMO) symposium, important further data supporting the benefit of immunotherapy in patients with upper gastrointestinal tumours were presented, along with encouraging activity of human epidermal growth factor receptor 2 (HER2) targeting drugs, both as monotherapy and in combination with immune checkpoint inhibitors. CheckMate 649 (LBA7),^1^ a global, open-label, randomized phase III trial including 2031 patients with unresectable gastric/gastroesophageal junction (GEJ) or oesophageal adenocarcinoma already changed clinical practice and led to Food and Drug Administration approval of nivolumab with fluoropyrimidine-based and platinum-containing chemotherapy, for all randomised patients and those with a combined positive score (CPS) ≥5, and *European Medicines Agency* approval for patients with CPS ≥5, respectively. At ESMO 2021, long-term follow-up (minimum 24 months for the nivolumab plus chemotherapy arm and 35.7 months for the nivolumab plus ipilimumab arm) and microsatellite-instability-high (MSI-H) tumours had been reported. Improved benefits from the addition of nivolumab to chemotherapy in all randomized patients [hazard ratio (HR) for overall survival (OS) 0.79; 95% confidence interval (CI) 0.71-0.88] and patients with programmed death-ligand 1 (PD-L1) CPS ≥5 (HR for OS 0.70, 95% CI 0.61-0.81) were confirmed. Again in patients with MSI-H tumours, the HR was in favour of nivolumab-treated patients with 0.38 (0.17-0.84), defining this excellent combination for first-line treatment of advanced MSI-H gastric cancer. In addition, new results for patients treated with nivolumab 1 mg/kg and ipilimumab 3 mg/kg followed by nivolumab 240 mg every 2 weeks without chemotherapy were presented. Herein, the data monitoring committee had closed the treatment arm with this combination of nivolumab and ipilimumab prematurely after inclusion of 409 patients. In both, the group of patients with a CPS ≥5 and all randomized patients, median survival and HRs demonstrated no significant benefit from this combination. Whereas, especially in patients with a CPS ≥5, an advantage in survival rates at 24 months (25 versus 17%) for patients treated with nivolumab and ipilimumab versus chemotherapy alone was demonstrated, patients living <1 year lived for a shorter time when treated without chemotherapy.

The importance of chemotherapy has also been confirmed for patients with HER2-positive advanced/metastatic gastric cancer in the randomized IIT phase II INTEGA (LBA54) AIO-trial,^2^ in which the combination of trastuzumab, nivolumab, and FOLFOX showed increased efficacy with 70% OS rate at 12 months. In the ToGA trial^3^ comparing trastuzumab plus chemotherapy with chemotherapy alone, the OS rate at 16 months was 55% and in the INTEGA chemotherapy-free arm, trastuzumab/nivolumab/ipilimumab did not improve the survival.

In the first read-out from the MAHOGANY trial, however, encouraging response rates for the chemotherapy-free combination of margetuximab (anti-HER2) and retifanlimab [anti-programmed cell death protein 1 (anti-PD-1)] with a confirmed overall response rate (ORR) rate of 53% were reported.^4^

Additionally, in two randomized, first-line phase III trials conducted in China (Orient-15^5^ and Orient-16^6^), the combination of sintilimab (anti-PD-1) plus chemotherapy demonstrated significant survival benefits compared with chemotherapy alone in metastatic/advanced patients with oesophageal squamous cell carcinoma (Orient-15, PD-L1 CPS ≥10, 17.2 months versus 13.6 months; *P* = 0.0018 and all patients, 16.7 months versus 12.5 months; *P* < 0.0001) and adenocarcinoma (Orient-16, PD-L1 CPS ≥5, 18.4 months versus 12.9 months; *P* = 0.0023 and all patients, 15.2 months versus 12.3 months; *P =* 0.009).

So far, the promise of chimeric antigen receptor T-cell (CAR-T cell) therapy has not yet been realized in solid tumours, due to historically low response rates and concerns regarding on-target, off-tumour toxicities. Claudin 18.2, as a tight junction protein which is specifically expressed on tumours of the upper gastrointestinal tract, with very limited expression elsewhere in the body, renders it an attractive target for CAR-T-cell therapy. In a single-centre Asian study,^7^ CAR-T cells targeting claudin 18.2 demonstrated encouraging efficacy signs with limited toxicity. In a cohort of chemorefractory gastrointestinal tumours the ORR was 48.6% (18/37), whereas in a subset of gastric and gastroesophageal adenocarcinoma patients the ORR was 61.1%. Toxicity was manageable, cytokine release syndrome was observed in 35 patients (94.5%), with all events only grade 1/2. Progression-free survival (PFS) was 5.6 months (95% CI 2.6-9.2 months) in patients treated at the recommended phase II dose in the gastric/GEJ cohort, with a relatively short follow up of 7.6 months (95% CI 5.6-8.6 months). The efficacy of claudin 18.2 CAR-T cells was not influenced by disease histology or previous immunotherapy use, but was affected by CAR-T cell peak levels. These data are quite encouraging but will require validation in further trials and in non-Asians.

In other ‘non-immunotherapy studies’, the DESTINY Gastric-02 study (LBA55)^8^ evaluated the antibody drug conjugate trastuzumab deruxtecan in retained HER2-positive advanced gastroesophageal cancer patients previously treated with chemotherapy plus trastuzumab. In the previously presented DESTINY Gastric-01 study,^9^ trastuzumab deruxtecan had demonstrated efficacy in Asian HER2-positive gastric cancer patients but had not yet been evaluated in non-Asian patients. The single-arm Gastric-02 second-line trial had a confirmed ORR of 38% to trastuzumab deruxtecan, also comparable to DESTINY Gastric-01, with an encouraging duration of response of 8.1 months (95% CI 4.1 months-NA). Importantly, significant numbers of drug-induced pneumonitis were not observed.

## Lower gastrointestinal tract

### Task force Colon, Rectum, Anal canal, chairs: Mark Peeters, Thibaud Koessler, Francesco Sclafani, Dirk Arnold, Lucjan Wyrwicz

#### Localised rectal cancer

As yet, the role of neoadjuvant chemotherapy as a single modality in rectal cancer has not been precisely established. Only limited data from the randomized phase II study, FOWARC,^10^ suggested it may lead to similar outcomes as chemoradiation. In the CONVERT study, presented by Pei-Rong Ding et al.,^11^ 663 patients with locally advanced rectal cancer with uninvolved mesorectal fascia-negative were randomized to four neoadjuvant cycles of CapeOx (nCT) or chemoradiation with concurrent capecitabine (nCRT). The early outcomes for both arms were similar, although nCRT was superior in the degree of tumour regression calculated as TRG 0-1 (38.6% for nCRT versus 24.0% for nCT; *P* < 0.001) with similar pathological complete responses rates (13.8% for nCRT and 11.0% for nCT; not significant). Sphincter preservation and R0 resection rates were also equal in both arms. The only parameters favouring nCT were the numbers of perioperative distant metastases (0.7% in nCT versus 3.1% in nCRT; *P* = 0.034) and the fraction of patients with preventive diverting ileostomy (52.2% in nCT versus 63.6% in nCRT; *P* = 0.008). Thus, these early CONVERT data do not support changes for rectal cancer treatment but confirm neoadjuvant chemotherapy as an option for patients with contraindications to radiotherapy.

#### Metastatic colorectal cancer (phase II and III)

In 218 first-line, mismatch repair (MMR)-unselected, metastatic colorectal cancer (mCRC) patients, immunotherapy (atezolizumab, anti-PD-L1) with triplet chemotherapy plus a biologic agent was studied in the randomized, phase II AtezoTRIBE trial,^12^ comparing folinic acid, fluorouracil, irinotecan and oxaliplatin (FOLFOXIRI)-bevacizumab (eight or more cycles) followed by maintenance [5-fluorouracil (5-FU)/leucovorin-bevacizumab] with FOLFOXIRI-bevacizumab-atezolizumab followed by maintenance (5-FU/leucovorin-bevacizumab-atezolizumab). At progression, FOLFOXIRI-bevacizumab with or without atezolizumab was reintroduced. The primary endpoint was PFS. Adding atezolizumab increased median PFS from 11.5 to 13.1 months (HR 0.69, 80% CI 0.56-0.85; *P* = 0.012) in the entire population, with this marginal benefit likely to be driven by MSI tumours. In fact, in the subgroup of patients with pMMR tumours (93%), median PFS was 11.4 months for FOLFOXIRI-bevacizumab and 12.9 months for FOLFOXIRI-bevacizumab-atezolizumab (HR 0.78, 80% CI 0.62-0.97; *P* = 0.071). Both arms had a similar ORR (64% versus 59%). OS data were not mature yet, with only 28% of events having occurred. Frequency of grade 3/4 adverse events was similar in both arms, with neutropenia being the most common toxicity. Immune-related adverse events were more common with atezolizumab (3% versus 1%), with transaminase increases being the most frequent.

The question of liver-directed treatment with systemic therapy was addressed by the international, multicentric, open-label, randomized (1 : 1) phase III EPOCH trial^13^ comparing radioembolization (yttrium-90) plus chemotherapy versus chemotherapy alone in unresectable liver metastases of second-line mCRC patients. Presence of unequivocal extrahepatic disease was an exclusion criterion. Primary endpoints were PFS and hepatic PFS (hPFS), according to blinded independent central review. Amongst 428 patients, >80% had bilobar disease and liver tumour burden was <10% in >50% of patients. Primary tumours were left-sided in 70% and 64% in the experimental and control arms, respectively. Sixty percent of patients received irinotecan-based second-line chemotherapy. Median PFS was 8.0 months with yttrium-90 chemotherapy versus 7.2 months with chemotherapy only (HR 0.69, 95% CI 0.54-0.88; *P* = 0.0013), whereas median hPFS was 9.1 versus 7.2 months (HR 0.59, 95% CI 0.46-0.77; *P* < 0.0001). OS was similar in both groups (14.0 versus 14.4 months). ORR appeared higher in the experimental arm (34% versus 21.1%). In the yttrium-90 chemotherapy arm, 55% had adverse device events, 10.7% with serious treatment-emergent adverse events (fatal in 4.3% of cases). These results are in line with those from FOXFIRE, SIRFLOX, FOXFIRE-Global and their pooled analysis and showed no OS benefit.

#### mCRC (early phase)

O6-Methylguanine DNA methyltransferase (MGMT) repairs DNA damage induced by alkylating agents such as temozolomide (TMZ). Secondary resistance to TMZ within MGMT-silenced tumours may be associated with a hypermutated status, frequently coupled with acquired mutations in MMR genes. MAYA^14^ was a proof-of-concept phase II trial in chemorefractory microsatellite stable (MSS), MGMT-silenced, mCRC. After two cycles of TMZ (150 mg/m^2^ daily for 5 days every 4 weeks), patients who had not progressed were treated with TMZ plus nivolumab and ipilimumab. The primary endpoint was 8-month PFS rate. Out of 703 screened patients, 142 (29%) were molecularly eligible and enrolled. Of these, 24% did not experience early progression and entered the second immunotherapy part. At 8 months, the median PFS rate was 36%. Median OS was 18.4 (14-NA) months, while ORR was 42%, showing promising results in a highly selected subgroup.

KRAS^G12C^ mutations occur in 3%-4% of all mCRC tumours. After the first results on KRAS^G12C^ inhibition in mCRC during last year’s ASCO with the multicohort CodeBREAK 100 study for sotorasib,^15^ the phase Ib/II trial KRYSTAL-1 investigators^16^ presented adagrasib, a selective and irreversible inhibitor of KRAS^G12C^, combined with the epidermal growth factor receptor inhibitor cetuximab (*n* = 32, phase Ib) or as monotherapy (*n* = 46: mostly phase II) in unresectable mCRC. Primary endpoints for phase I were safety, maximum tolerated dose, pharmacokinetics, recommended phase II dose and ORR in phase II. Median follow-up was 7 and 8.9 months in the combination and monotherapy group, respectively. Patients were heavily pretreated in both groups, with more than three prior lines in 55%-65%. Response rates were 22% (monotherapy) and 43% (combination); disease control rate (DCR) 87% and 100%, respectively. These results compare favourably with the 15.4% confirmed ORR with sotorasib and panitumumab in CodeBreaK 101.^17^ In KRYSTAL-1, treatment was generally well tolerated, with grade 3/4 treatment-related adverse events <5%, 12.9% in CodeBreaK 101. Adagrasib plus cetuximab is now being evaluated in the 2L phase III KRYSTAL-10 study (NCT04793958). Further combinations of interest are those with a downstream MEK inhibitor, as well as with an inhibitor of the RAS activation factor, SOS.

Agents targeting the DNA damage repair (DDR) pathway have been successful in tumours with DDR pathway alterations. WEE1 plays a central role in cell cycle regulation and genomic stability, whereas WEE1 inhibition induces DNA damage and DNA replication stress. Preclinical models harbouring *RAS*-positive *TP53*-mutant mCRC tumours generally produce G1/S checkpoint failure. Thus, a WEE1 inhibitor may lead to impaired checkpoint control and DNA replication stress. In the multicohort FOCUS4-C maintenance study,^18^ *RAS*-positive TP53-mutant patients without disease progression after 16 weeks of standard chemotherapy induction were randomized to either active monitoring (*n* = 25) or the WEE1 inhibitor adavosertib (*n* = 44). The primary endpoint, PFS, was significantly improved (HR 0.35, 95% CI 0.18-0.68; *P* = 0.0022), although median PFS was rather short in both arms (1.87 versus 3.61 months, respectively). According to an exploratory analysis, this effect was more pronounced in left-sided tumour primaries (HR left/right sidedness: 0.24/1.02).

The antibody/drug conjugate trastuzumab deruxtecan had remarkable activity in patients with unresectable or mCRC, showing HER2 expression and *RAS*/*BRAF*^*V600E*^ wild-type status, even after at least two prior regimens, in the multicentre phase II DESTINY-CRC01 trial: Siena et al.^19^ recently reported an ORR of 45.3% and a DCR of 83% in HER2+ immunohistochemistry 3+ (IHC3+) or IHC2+/ISH+ patients (*n* = 53) with trastuzumab deruxtecan. The corresponding median PFS and OS were 6.9 and 15.5 months, respectively. The biomarker analysis at ESMO 2021 suggests an association between baseline HER2 expression levels, as the median PFS of IHC3+ patients (*n* = 40; 8.3 months) was markedly longer than those with IHC2+/ISH+ status (*n* = 13; 4.1 months). This was the strongest prognostic denominator, amongst many exploratory factors, including plasma cERBB2 amplification status, plasma *RAS*- and *BRAF*-mutation and plasma PIK3CA gain-of-function mutation status. The ctDNA analysis suggested relevant antitumour activity in patients with HER2+ mCRC who have *RAS*- or PIK3CA-activating mutations or higher baseline TMB levels. All these cautious conclusions, given the limited sample size, will be undergoing confirmation in the upcoming DESTINY-CRC02 trial (NCT04744831).^20^

## Hepatobiliary and pancreatic cancer, neuroendocrine tumours

### Task force Hepatobiliary, NETs, chairs: Juan W Valle, Jens Ricke

Recent years in biliary tract cancer (BTC) have been marked by the advent of routine molecular screening and personalized therapy for specific patients with *IDH1* mutation, *FGFR2* fusions, *BRAF* mutations, HER2 alterations, or MSI.^21^ This ESMO congress provided new data regarding chemotherapy in non-molecularly selected BTC as Perkhofer et al.^22^ presented the randomized, non-comparative phase II NIFE AIO trial, which evaluated first-line 5-FU/leucovorin plus nanoliposomal irinotecan (nal-iri). Of note, nal-iri with 5-FU/leucovorin was approved for metastatic pancreatic cancer based on positive survival of the NAPOLI-1 phase III trial.^23^ The randomized, phase II NIFTY trial, presented at ASCO 2021, reported first results of this combination chemotherapy in second-line Asian cholangiocarcinoma carcinoma (CCA) patients, with improved PFS, OS, and ORR versus 5-FU/leucovorin alone.^24^

In the NIFE AIO trial, chemotherapy-naive, intra- or extrahepatic CCA patients with Eastern Cooperative Oncology Group performance status 0-1 received standard cisplatin plus gemcitabine (CISGEM) or the 5-FU/leucovorin plus nal-iri combination. The primary endpoint was PFS at 4 months, with a fixed PFS rate of interest of ≥50% in the intention to treat (ITT) population. The primary endpoint was met for 93 patients with a 4-month PFS rate of 51% with 5-FU/leucovorin plus nal-iri (59.5% with CISGEM). Median PFS was 6.0 months (95% CI 2.4-9.6 months) with 5-FU/leucovorin plus nal-iri and 6.9 months (95% CI 2.5-7.8 months) with CISGEM. Median OS (data not mature) was 15.9 months (95% CI 10.6-21.8 months) and 13.6 months (95% CI 6.5-17.7 months), and ORR was 24.5% and 11.9%, respectively. Interestingly, a differential effect was observed according to primary tumour location, with median PFS in intrahepatic CCA of 3.5 months (95% CI 2.1-6.0 months) with 5-FU/leucovorin plus nal-iri versus 7.7 months (95% CI 6.0-9.5 months) with CISGEM (median OS 14.2 versus 16.4 months, *n* = 66), and 9.6 months versus 1.8 months in extrahepatic CCA (median OS 18.2 versus 6.3 months, *n* = 25). Overall, this trial met its prespecified threshold of activity, providing new data for the use of nal-iri in BTC, particularly in extrahepatic CCA. With the limited sample size, however, this differential effect between intra- and extrahepatic CCA remains to be confirmed and the underlying biological mechanisms to be explored. Thus, these results are not practice changing and more data are needed. Further results of the randomized phase II AIO NALIRICC trial (NCT03043547), which evaluates 5-FU/leucovorin plus nal-iri combination versus 5-FU/leucovorin alone after gemcitabine-based first-line chemotherapy (i.e. the same design as the NIFTY trial but in a non-Asian population), are awaited.

The academic First International Randomized phase II Study in Malignant Progressive Pheochromocytoma and Paragangliomas (FIRSTMAPP) study^25^ randomized 78 patients with these very rare tumours with strong expression of vascular endothelial growth factor (VEGF), platelet-derived growth factor (PDGF) and VEGF receptor (VEGFR)-1,2 and PDGF receptor (PDGFR), to sunitinib [37.5 mg once daily shown to be effective in pancreatic neuroendocrine tumours (NETs^26^)] or placebo. The study met its primary endpoint with a 12-month PFS (centrally reviewed) of 35.9% on sunitinib (18.9% in the control arm, which included 20% in the 95% CI, confirming the statistical assumption). The median PFS was 8.9 months (95% CI 5.5-12.7 months) with sunitinib versus 3.6 months (95% CI 3.1-6.1 months) with placebo. No new toxicity concerns emerged, despite hypertension being an issue in patients with phaeochromocytoma and paragangliomas. This is a first practice-changing study in this patient group, previously thought to be too rare, also as treatment options are limited for these rare cancers.

The phase III SPINET study^27^ evaluated the activity of somatostatin analogue lanreotide in patients with advanced well-differentiated bronchopulmonary NETs. Seventy-seven patients were randomized (2 : 1) to lanreotide or placebo, stratified by tumour grade: typical versus atypical carcinoid. As the study closed early due to slow accrual, the primary endpoint, median PFS, was 16.6 months (95% CI 11.3-21.9 months) although patients with typical carcinoids derived greater benefit from lanreotide (21.9 months versus 13.9 months with placebo) than those with atypical carcinoids (13.8 months versus 11.0 months with placebo), in keeping with findings of lanreotide^28^ or octreotide^29^ in well-differentiated gastrointestinal NETs.

The phase II/III AXINET study provided an updated blinded central review of PFS in advanced well-differentiated (grade 1-2) extra-pancreatic NET patients treated with a somatostatin analogue (octreotide LAR) with either axitinib or placebo. The results of the investigator-assessed primary endpoint PFS were previously presented at ASCO-GI 2021^30^: median PFS 17.2 months versus 12.3 months (HR 0.816; *P* = 0.169). The robust method of blinded central radiological assessment (secondary endpoint) determined the PFS as 16.6 months versus 9.9 months for axitinib and placebo, respectively (HR 0.687, *P* = 0.01).^31^ This clinically relevant difference is in keeping with the effects of VEGF inhibition in pancreatic NET^26^ and the FIRSTMAPP study detailed above.^25^

## Pancreatic cancer

### Task force Pancreas, chair: Cindy Neuzillet

Data on perioperative chemotherapy in resectable pancreatic ductal adenocarcinoma (PDAC) are limited. The PREOPANC phase III study compared immediate surgery followed by adjuvant gemcitabine and chemoradiotherapy with gemcitabine followed by surgery and adjuvant gemcitabine, versus upfront surgery followed by adjuvant gemcitabine, in a mixed population of borderline and resectable tumours.^32^ The trial initially negative, now showed an OS improvement, but only on its updated long-term follow-up OS data in the overall population (ASCO 2021),^33^ with no benefit in the resectable PDAC subgroup.

Seufferlein et al.^34^ presented the non-comparative, randomized, phase II NEONAX trial with perioperative versus adjuvant chemotherapy of gemcitabine/nab-paclitaxel in patients with resectable PDAC. Of note, this chemotherapy regimen failed to show its superiority over surveillance in the previous phase III APACT study.^35^ In NEONAX, 127 patients with resectable PDAC were randomized 1 : 1 between perioperative gemcitabine plus nab-paclitaxel (two preoperative and four post-operative cycles, arm A) or adjuvant treatment (six cycles, arm B). Two populations were defined: ITT corresponding to patients fulfilling the inclusion criteria (*n* = 59 in both arms), and modified ITT (mITT) comprising patients from the ITT population who either completed neoadjuvant therapy and underwent surgery (R0/1 resection) in arm A, or patients after surgery (R0/R1 resection) having started adjuvant therapy (at least one cycle) in arm B. The primary objective was improved DFS at 18 months from 38% to 55% in the mITT population. The ORR in arm A was 28.9%, with a low rate of progression during neoadjuvant chemotherapy of 6.7%. The resection rate was 69.5% in arm A and 78.0% in arm B, with more R0 resections in arm A (87.8% versus 67.4%). In the ITT population, the 18-month PFS rate was 28.7% in arm A versus 19.3% in arm B, and the median PFS was 11.4 months versus 5.9 months. The 18-month PFS rate in mITT was 32.2% in arm A (*n* = 39, 66.1% of ITT patients) and 41.4% in arm B (*n* = 25, 42.4%), however, not reaching the prespecified target in both arms, and the median DFS was 14.1 months and 17.0 months, respectively. Thus, perioperative chemotherapy was safe, but did not achieve the expected 18-month PFS rate of 55% in the mITT population. The definition of this population was questionable, however, and the analysis in the ITT population (considered as more clinically relevant) favoured perioperative chemotherapy. Even if negative, this study reinforces the rationale for neoadjuvant chemotherapy in resectable pancreatic cancers, pending the results of the PANACHE-01 (NCT02959879) and ALLIANCE 021806 (NCT04340141) randomized studies evaluating perioperative versus adjuvant chemotherapy with FOLFIRINOX.

The phase III PRODIGE 24 study demonstrated superiority of modified FOLFIRINOX (mFOLFIRINOX) compared with gemcitabine as adjuvant chemotherapy for resected PDAC on DFS (primary endpoint).^36^ At the initial publication, OS data were not mature and prognostic factors for OS could not be analysed. At ESMO 2021, updated 5-year OS results with median follow-up of 69.7 months and the analysis of prognostic factors were presented by Conroy et al.^37^ The median OS was 53.5 months (95% CI 43.5-58.4 months) with mFOLFIRINOX versus 35.5 months (95% CI 30.1-40.3 months) with gemcitabine, with a stratified HR of 0.68 (95% CI 0.54-0.85; *P* = 0.0009). The 5-year OS rates were 43.2% (95% CI 36.5% to 49.7%) and 31.4% (95% CI 25.5% to 37.5%), respectively. Specific survival (HR 0.65, 95% CI 0.51-0.82, *P* = 0.0003) and metastasis-free survival (HR 0.64, 95% CI 0.52-0.80, *P* = 0.0001) also improved with mFOLFIRINOX. Treatment arm, tumour grade and stage, and patient age were independent prognostic factors for OS; volume of the centres was not retained. In addition, completion of all 12 treatment cycles (regardless of duration and dose intensity) was also associated with a more prolonged OS (HR 0.64, 95% CI 0.49-0.84, *P* = 0.002). Thus, these updated results of PRODIGE 24/CCTG PA6 confirm adjuvant mFOLFIRINOX as standard of care in PDAC. Completion of adjuvant therapy is also an important prognostic factor, already shown in the ESPAC-3 study.^38^

## Early translational phase trials

### Task Force for individualized cancer therapy, chairs: Radka Obermannová, Maria Alsina

Metastatic MSI-H/dMMR gastrointestinal tract cancers (GITC) should be addressed for first-line immunotherapy, according to different presentations at ESMO 2021,^39^^,^^40^ clearly further supporting this clinical unmet need. A phase II study evaluating the efficacy of neoadjuvant pembrolizumab in localized MSI-H/dMMR solid tumours^39^ enrolled mostly colorectal (*n* = 27), pancreatic (*n* = 2), and duodenal (*n* = 2) cancers. High response rates were observed with ORR of 75% and pCR of 69% in patients undergoing surgical resection. The already reported frequency of MSI-H nonmetastatic esophagogastric adenocarcinoma^41^ had been verified in the phase II DANTE trial^42^ and at the EURECCA database at Vall d’Hebron University Hospital. The DANTE trial added atezolizumab to perioperative fluorouracil, leucovorin, oxaliplatin, and docetaxel (FLOT). High frequency of pCR (38.5%) was described in 5 of 23 MSI-H tumours treated with atezolizumab.^42^^,^^43^ The EURECCA database identified 12 of 92 (13%) MSI-H patients with local and locally advanced oesophagogastric cancers.^44^

Cholangiocarcinoma is a rare but heterogeneous disease with a frequency of ∼3% of gastrointestinal tumours.^45^ Several molecular pathways including mutations in *IDH1*, *IDH2*, *BRAF*, *PI3K*, *MET*, or translocations in *FGFR2* are targetable. In the upcoming PAMICC study, EORTC addresses the role of IDH1 and HRD mutations inducing a homologous recombination repair defect which increases tumour sensitivity to poly(ADP-ribose) polymerase (PARP) inhibition (NCT04796454).^46^ At ESMO 2021, the 334TiP trial is a phase I trial of dual-targeted drugs niraparib (PARP inhibitor) plus anlotinib (VEGFR, FGFR, PDGFR, and c-kit inhibitor) in homologous recombination repair (HRR) gene-mutated advanced solid tumours including BTC.^47^ FGFR2 fusions/rearrangements (FGFR2^fus^ in ∼15% of intrahepatic cholangiocarcinoma) were addressed in FIDES-01.^48^ Derazantinib (FGFR1-3 inhibitor), in the FGFR2^fus+^ cohort (*n* = 103), showed encouraging efficacy and met its primary endpoint with a centrally confirmed ORR of 21.4%. (95% CI 13.9% to 30.5%), mPFS 8.0 months (95% CI 5.5-8.3 months), and mOS 15.5 months (95% CI 12.5-22.6 months).

Based on the results of MOUNTAINEER (NCT03043313) in HER2-positive mCRC, the ongoing dose escalation and expansion phase Ib/II SGNTUC-024 (NCT04430738) trial evaluates the efficacy of tucatinib (HER2 inhibitor) plus trastuzumab plus FOLFOX in patients with HER2-positive gastric, oesophageal, and GEJ adenocarcinoma, cholangiocarcinoma, gallbladder carcinoma, and CRC.^49^

## Discussion

During the ESMO 2021 Congress, we and other academic groups did not present any practice-changing trials in advanced gastrointestinal cancers. Important proof-of-concept studies, however, suggest new possible future treatment avenues, including cellular therapies in upper gastrointestinal malignancies and chemotherapy-immunotherapy combinations in historically immune-refractory MSS colorectal cancers. Whereas anti-PD1 inhibitors continue to improve outcomes in several subgroups of gastrointestinal tumours and in different indications, some drawback signals for immune checkpoint combination strategies, with anti-CTLA4 as an example, are now coming to light such as in the CheckMate 649 trial or in the INTEGA trial for instance.

Chemotherapy dependency as a one-size-fits-all approach maintains its validity in several gastrointestinal cancers and continues to be successfully explored, especially in academic trials and in difficult to treat indications, like the practice-changing PRODIGE-24 study in pancreatic cancers or the newly reported NIFE AIO trial in BTCs. Conversely, precision medicine approaches with targeted agents continue to evolve and explore molecularly selected subgroups of patients with tumours carrying very rare molecular events, with frequency below 5%, but potentially very effective. Such personalized approaches based on genomic testing are changing daily oncology practice. Initially, Gunnar Folprecht and our GITCG group started the EORTC SPECTACOLOR platform for CRC already in 2015: https://www.eortc.org/blog/2015/01/14/spectacolor-viable-next-generation-multinational-cancer-clinical-trial-infrastructure/. Meanwhile, a new improved example is the German’s Platform for Analyzing Targetable Tumour Mutations (PLATON): a permanently open, multicenter, prospective, cohort study including bio-banking and sharing the platform infrastructure for associated substudies.^50^ The platform PLATON focuses on GITC tumour molecular profiling and offers clinical investigators to select optimal clinical trials based on their molecular profile. An interactive web application in a virtual Molecular Tumour Board is also available and has already analysed 75 patients.

This is why continuous efforts in maintaining fruitful interactions between academia, Pharma, and new cutting-edge technologies of diagnostic providers are essential for the successful development of new drugs and strategies to ultimately improve clinical outcomes for our cancer patients. We as the EORTC Gastrointestinal Tract Cancer Group welcome further interactions between academic key opinion leaders, national groups, active clinicians and scientists across Europe, pharma-companies, and biotech partners, to foster the development of innovative, multidisciplinary, and patient-centred ideas for future practice-changing trials.
